# New Insight on Water Status in Germinating *Brassica napus* Seeds in Relation to Priming-Improved Germination

**DOI:** 10.3390/ijms20030540

**Published:** 2019-01-28

**Authors:** Katarzyna Lechowska, Szymon Kubala, Łukasz Wojtyla, Grzegorz Nowaczyk, Muriel Quinet, Stanley Lutts, Małgorzata Garnczarska

**Affiliations:** 1 Department of Plant Physiology, Institute of Experimental Biology, Faculty of Biology, Adam Mickiewicz University, Poznań, ul. Umultowska 89, 61-614 Poznań, Poland; katarzyna.lechowska@amu.edu.pl (K.L.); szymon.globus@ibb.waw.pl (S.K.); lukasz.wojtyla@amu.edu.pl (Ł.W.); 2 NanoBioMedical Centre, Adam Mickiewicz University, Poznań, ul. Umultowska 85, 61-614 Poznań, Poland; grzegorz.nowaczyk@amu.edu.pl; 3 Groupe de Recherche en Physiologie Végétale (GRPV), Earth and Life Institute–Agronomy (ELI-A), Université catholique de Louvain, Croix du Sud 45, boîte L7.07.13, B-1348 Louvain-la-Neuve, Belgium; muriel.quinet@uclouvain.be (M.Q.); stanley.lutts@uclouvain.be (S.L.)

**Keywords:** seed priming, germination, water status, aquaporin genes, NMR spectroscopy, SEM microscopy, TEM microscopy

## Abstract

Seed priming is a pre-sowing method successfully used to improve seed germination. Since water plays a crucial role in germination, the aim of this study was to investigate the relationship between better germination performances of osmoprimed *Brassica napus* seeds and seed water status during germination. To achieve this goal, a combination of different kinds of approaches was used, including nuclear magnetic resonance (NMR) spectroscopy, TEM, and SEM as well as semi-quantitative PCR (semi-qPCR). The results of this study showed that osmopriming enhanced the kinetics of water uptake and the total amount of absorbed water during both the early imbibition stage and in the later phases of seed germination. The spin–spin relaxation time (*T*_2_) measurement suggests that osmopriming causes faster water penetration into the seed and more efficient tissue hydration. Moreover, factors potentially affecting water relations in germinating primed seeds were also identified. It was shown that osmopriming (i) changes the microstructural features of the seed coat, e.g., leads to the formation of microcracks, (ii) alters the internal structure of the seed by the induction of additional void spaces in the seed, (iii) increases cotyledons cells vacuolization, and (iv) modifies the expression pattern of aquaporin genes.

## 1. Introduction

Effective seed germination is of great importance in agriculture. The rapid and uniform seedling emergence is the basic requirement to increase crop yield and quality. The genus *Brassica* includes more than 30 species plus several varieties and hybrids. Among these are several important species in agriculture, used for human consumption, animal fodder and non-food industrial uses. *Brassica napus* is one of the world’s most important vegetable oil sources and at the same time the most productive species of *Brassica* oilseed crops [1], making it the crucial plant for agriculture. Seed germination begins with the process of water uptake by the mature seed called imbibition [2]. Besides oxygen and the right temperature, the inflow of water to the non-dormant quiescent dry seed is the primary event required for the initiation and proper course of germination. Water is necessary for the resumption of metabolic activity since it creates the environment for biochemical reactions, stabilizes the conformation of macromolecules and acts as a reactant in numerous processes [3]. The restoration of metabolic activity, together with appropriate turgor pressure generated by the progressively increasing water content, leads to embryo growth and ultimately to radicle protrusion through the seed coat, which defines the endpoint of germination. 

The rate and level of seed imbibition are controlled by three main factors: seed composition, water availability in medium and seed coat water-permeable properties [3]. The seed coat is an outer layer in the seed structure that plays a fundamental role in managing the interactions between the embryo and the external environment, controlling the seed development and seed filling, protecting the embryo against adverse biotic and abiotic factors, regulating gaseous exchange, and contributing to seed dispersion [4,5,6,7]. Seed coat water permeability is determined by its chemical composition and structural characteristics [8,9]. The seed coat may be either impermeable, acting as a key factor imposing physical seed dormancy in some species, or permeable to water, thus taking part in the regulation of the rate of water infiltration into the seed to a level that ensures efficient hydration but prevents the occurrence of imbibitional injuries [10]. Numerous studies indicated that, depending on the species, the micropylar region, hilum and/or lens, in the case of legume plants, are the major sites of water entry into the seed [11,12,13,14,15,16,17]. Recently, a magnetic resonance imaging (MIR) study showed that the entry point of water in seeds of *B. napus* is the small seed coat section abutting the hilum, thus supporting previous observations obtained for other plant species [18]. This study also revealed that, in *B. napus*, the swelling of the embryo is a non-uniform process that starts with the hydration of the radicle, triggering the rehydration of other embryo parts. 

In light of the fact that vacuoles serve as water storage and osmotic compartments, vacuolization represents an important process affecting the water status of germinating seeds [19]. Vacuoles are strongly involved in cell elongation as they are the source of turgor pressure in the cells [20]. The enlargement of the vacuole precedes cell elongation, which is considered a basic process of cell growth in germinating seeds since only cell enlargement, not cell division, is required for the embryo to complete germination through radicle emergence [21,22,23]. 

Germination involves the absorption and transportation of large amounts of water and aquaporins (AQPs), that form water channels in the membranes and facilitate intra- and intercellular water movement, appear to play a significant role in this process. Several studies indicated the possible contribution of AQPs to the regulation of water transport during seed germination [24,25,26,27,28,29]. AQPs belong to the major intrinsic protein (MIP) superfamily and are divided into five subfamilies [30]. The members of two subfamilies—plasma membrane intrinsic protein (PIPs) primarily localized in the plasma membrane and tonoplast intrinsic protein (TIPs) governing water fluxes across the vacuolar membrane—are considered essential players in transmembrane water transport [31]. Based on a phylogenetic analysis, PIPs are divided into two subgroups (PIP1 and PIP2), demonstrating the difference in water transport activity [32]. Moreover, they are capable of mutual interactions that result in the modification of their activity, trafficking, and gating [33]. 

Seed priming is a pre-sowing treatment successfully used to improve the germination performance and stand establishment of various cultivated plants in both optimal and adverse environmental conditions. The seed priming technique involves controlled seed hydration to an extent that enables the early seed germination events to begin but not to an extent that results in radicle protrusion. After partial imbibition, the seeds undergo the drying process to the original moisture levels without loss of viability and may be stored for relatively short time periods (up to several months) under favorable environmental conditions until needed [31].

There are several methods of seed priming developed in order to enhance the germination parameters and seedling vigor, including hydropriming, osmopriming, biopriming, solid matrix priming, hormonopriming, and others [31]. Osmopriming is a technique in which seed hydration during the soaking phase is restricted to the desired level by applying a solution with low osmotic potential [34]. Our previous studies revealed that osmopriming with polyethylene glycol (PEG) is a reliable method to advance the germination of *B. napus* seeds either in optimal or salt stress conditions [35,36]. Using a combined transcriptomic and proteomic approach, we have shown that the background of priming-improved germination is a highly complex process associated with the upregulation of genes and proteins involved in cell division, cell wall modification, cytoskeletal organization, oxidative stress response, as well as water transport [35]. Moreover, we demonstrated that osmoprimed *B. napus* exhibit enhanced tolerances to salt stress during germination due to an H_2_O_2_-mediated increase in the expression of the *P5CSA* gene and enzyme activity of its product, leading to the high accumulation of proline [36]. The efficiency of osmopriming with PEG was also confirmed by other authors in different plant species and stages of plant development, particularly under unfavorable environmental conditions [31,34].

Despite numerous studies focused on priming technology and the progress that has been made in recent years toward understanding the mechanisms underlying osmotic seed priming, our overall understanding of the physiological and biochemical changes responsible for the effects on germination, plant growth, and abiotic stress tolerance is still fragmentary. Water inflow to the seed is an essential prerequisite for the initiation and successful completion of the germination process; hence, the improved germination and growth parameters may be a result of the improved water uptake and more favorable water relations in primed seeds. The predominant feature of hydropriming is the increased seedling growth correlated with a higher water uptake by primed seeds [37]. Galhaut et al. [38] reported that priming with PEG and gibberellic acid (GA_3_) can hasten water uptake in *Trifolium repens* seeds. Moreover, the facilitation of water absorption and the enhancement of the germination potential evoked by priming treatments may be associated with seed coat and internal seed structure modifications, as well as with the upregulation of AQP genes [35,38,39,40]. Nonetheless, the differences in the water relations between primed and unprimed seeds and the reasons behind this are still unclear. The present study extends these previous studies by examining additional factors that could potentially affect the water relations and germination in primed *B. napus* seeds. For this purpose, different kinds of approaches were used, including nuclear magnetic resonance (NMR) spectroscopy, TEM, SEM, and semi-qPCR. These findings will provide a framework for integrating the structural, physiological, and metabolic events taking place during priming improved germination.

## 2. Results

### 2.1. Osmopriming Increases Seed Germination Performance and Water Uptake during Germination

**Figure 1:**
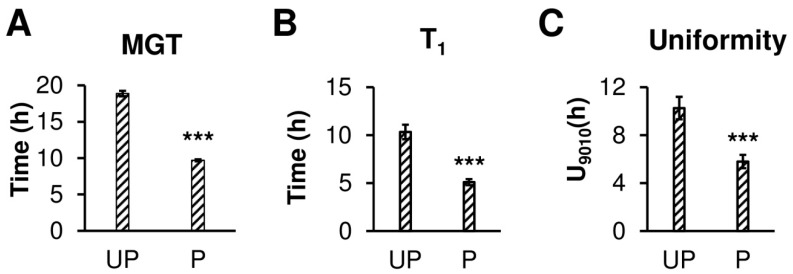
The germination parameters of dry unprimed (UP_d_) and primed dried (P_d_) *B. napus* seeds. Each value is a mean of 10 replicates (100 seeds per each), and the vertical bars denote SD. *** Significant differences are seen between means at *p* < 0.001 according to the *t*-test. (**A**) The mean germination time (MGT). (**B**) The time to reach 1% germination. (**C**) The uniformity of germination (U9010) against the time interval for between 90% and 10% of viable seeds to germinate.

**Figure 2:**
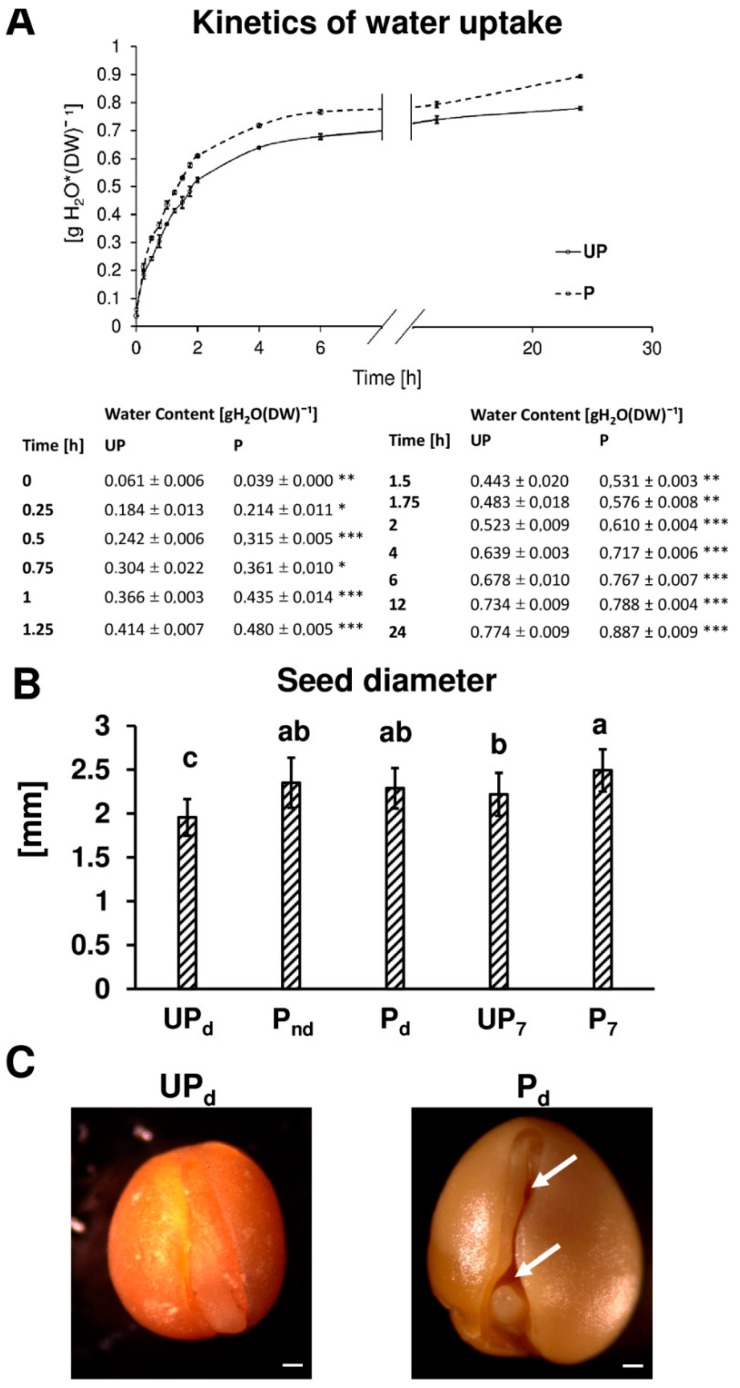
(**A**) The kinetics of water uptake during the germination of unprimed (UP) and primed (P) *B. napus* seeds. Detailed information is deposited below the chart; the differences between the means of the primed and unprimed seeds in each examined time point were statistically significant at *p* < 0.05 (*), *p* < 0.01 (**), or *p* < 0.001 (***) according to the *t*-test (*n* = 3). (**B**) The seed diameters of the unprimed and primed seeds before the start of germination (dry unprimed seeds, UP_d_; primed non-dried seeds, P_nd_; and primed dried seeds, P_d_) and during germination (unprimed seeds germinating 7 h, UP_7_; and primed seeds germinating 7 h, P_7_). Each value is a mean of 10 replicates (100 seeds per each), and the vertical bars denote SD. The differences were statistically significant, as determined by the one-way analysis of variance (ANOVA) and the Tukey–Kramer Multiple Comparison Test at *p* < 0.01. The same letters on the bars indicate insignificant differences between the means. (**C**) Photographs of the dry unprimed seeds (UP_d_) and primed dried seeds (P_d_) after removing the seed coat. The arrows indicate the enlarged void spaces between cotyledons and between each cotyledon and embryonic axis in the dry seeds after the osmopriming treatment. The bars represent 200 µm.

Our results indicate that the osmopriming treatment has a positive effect on the germination of *B. napus* seeds (Figure 1). Primed seeds germinated nearly twice as fast as unprimed ones (Figure 1A). Osmopriming caused a shift in the time required to reach 1% germination from 10 h to approx. 7 h (Figure 1B). Additionally, the primed seeds exhibit enhanced germination uniformity, according to the parameter U9010 that specifies the time interval for between 10% and 90% of viable seeds to germinate, showing a 1.8-fold lower value for primed seeds as compared to unprimed ones (Figure 1C). These results are in accordance with our previous works which showed that osmopriming promotes germination both in optimal conditions and under salt stress [35,36]. The kinetics of water uptake by primed and unprimed seeds during germination are illustrated in Figure 2A. Seed hydration was the most rapid at the first two hours of imbibition and started to slow down noticeably after 6 h of imbibition for both unprimed and primed seeds. However, primed seeds showed a higher rate of water uptake than unprimed ones during the early imbibition stage as well as in the later phases of germination. For primed seeds, the amount of water absorbed after 15 min, 30 min, 1 h, 6 h, and 24 h of imbibition was significantly higher as compared to the unprimed ones by 42%, 52%, 30%, 18%, and 19%, respectively (Figure 2A).

**Figure 3:**
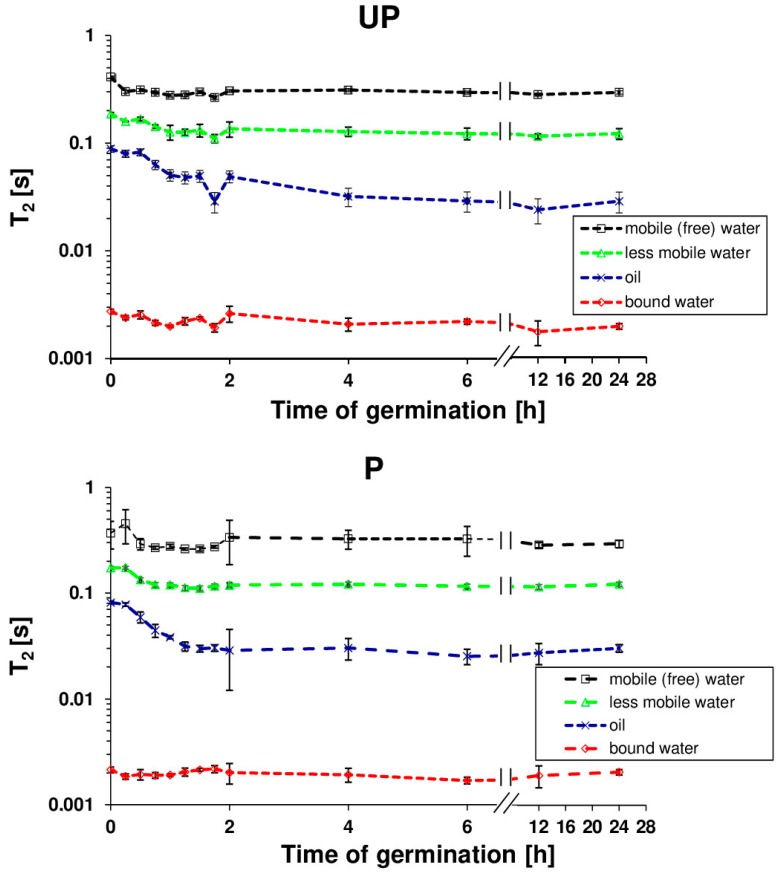
The components of the spin–spin relaxation (*T*_2_) in germinating primed (P) and unprimed (UP) *B. napus* seeds. Each value is a mean of three replicates, and the vertical bars denote SD.

**Figure 4:**
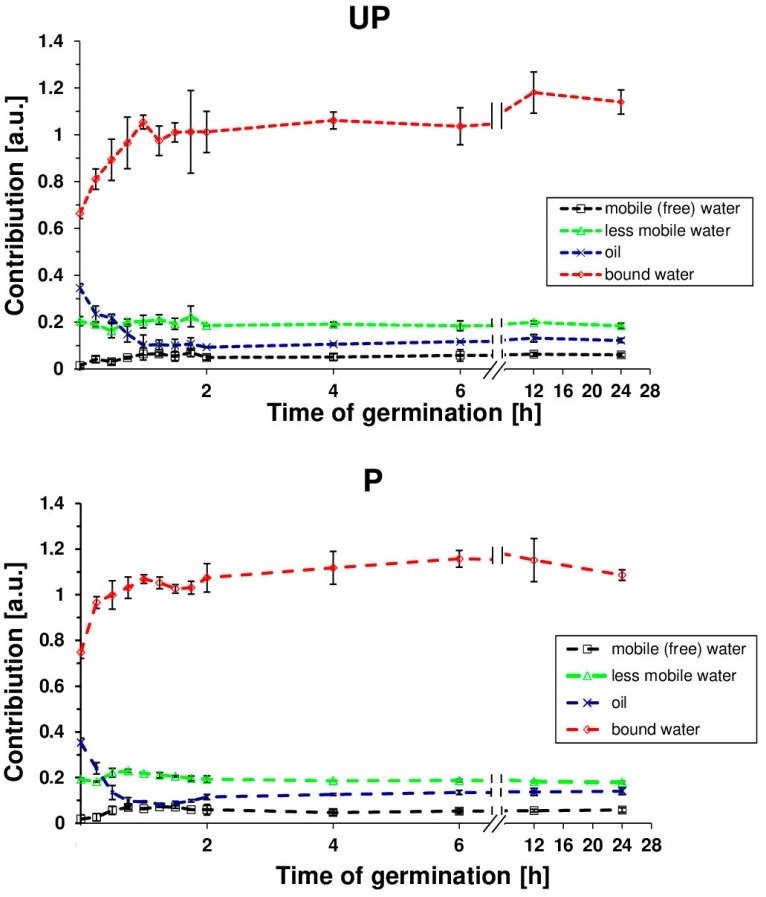
The contribution of various *T*_2_ relaxation time components of water during the germination of primed (P) and unprimed (UP) *B. napus* seeds. Each value is a mean of three replicates, and the vertical bars denote SD.

The seed hydration experiments did not shed light on the overall water penetration into specific seed compartments or structures. Therefore, for a detailed analysis of the state of water in *B. napus* seeds, the transverse relaxation time experiment was applied for primed and unprimed seeds as a function of hydration time. The spin–spin transverse relaxation time (*T*_2_) showed the existence of 3 water fractions in swelling seeds (Figure 3), each with different a magnetic environment causing a different relaxation rate. These populations may correspond to water molecules differing in mobility. The protons with the longest *T*_2_s (above 400 ms for dry seeds) were presumed to correspond to the mobile water fraction (free water); those with average *T*_2_s (approximately 200 ms for dry seeds) corresponded to the lower mobility fraction; and those with the shortest *T*_2_s (approximately 3 ms for dry seeds) corresponded to bound water. A fourth fraction was also identified with a *T*_2_ of 100 ms for dry seeds and was associated with the seed oil component. The highest changes in each component occurred within the first 2 h of imbibition with a rapid decline of the *T*_2_ values associated with oil fraction in primed seeds. Additional data (Figure 4) that concern the contribution of particular fractions in the total pool of water showed that the dominant component during the imbibition of *B. napus* seeds is the population of bound water. The dynamics of bound water for primed and unprimed seeds over the first 2 h reflects the difference in hydration levels of both primed and unprimed seeds.

### 2.2. Osmopriming Changes the Microstructural Features of the Seed Coat 

**Figure 5:**
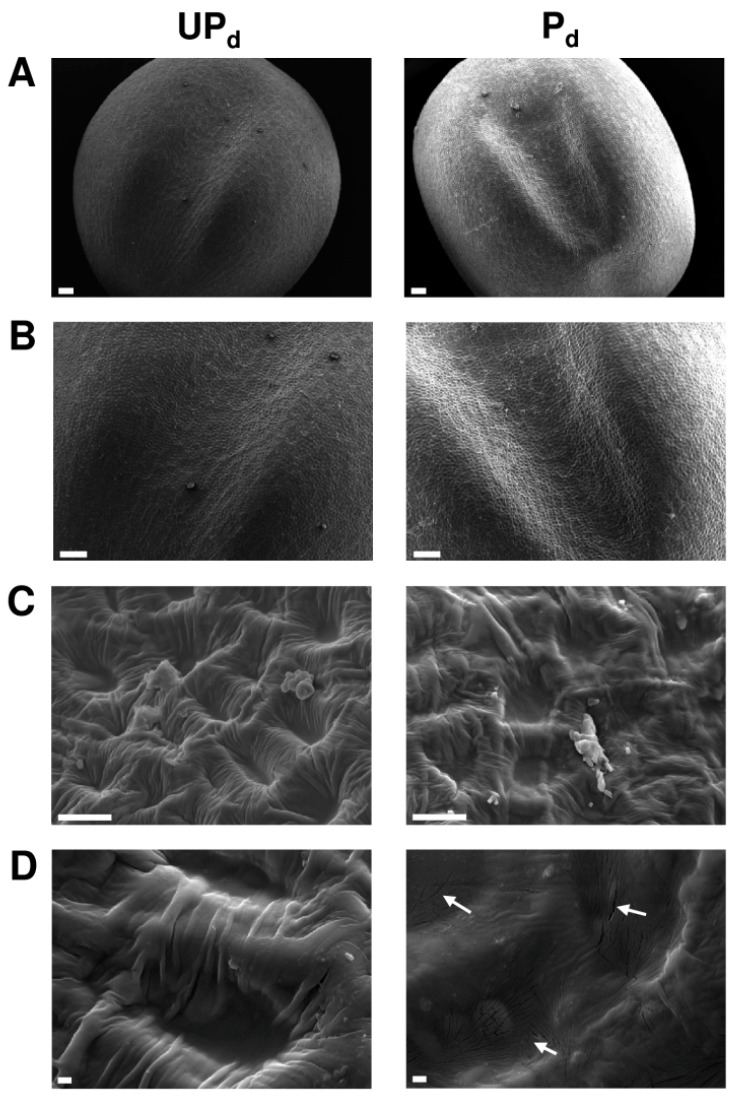
SEM images of the seed coat structure at the ventral side of the dry unprimed (UP_d_) and primed dried (P_d_) *B. napus* seeds at different magnifications. The bars represent (**A**,**B**) 100 µm, (**C**) 10 µm, and (**D**) 1 µm. Note the microcracks (arrows) inside the pits visible at the highest magnification (D) on the surface of the dry primed seeds.

Representative scanning electron micrographs show the seed coat structural characteristics of primed dried and dry unprimed seeds (Figure 5). According to the terminology proposed by Murley [41] and Zeng et al. [42] concerning seed coat microsculpture, both primed and unprimed seeds possess surface patterns belonging to the reticulate type. The surface sculpture is made up of irregular polygons with poorly developed outer anticlinal cell walls surrounding the interspace. In dry unprimed seeds, the reticulum interspace contains a daughter reticulation with a high and wide reticulum wall, irregularly formed pits, and undulations traversing the interspace. In primed dried seeds the reticulum interspace is smoother, flatter, and marked by multi-microcracks, located mainly inside the pits. Interestingly, microcracks are present in the testa regions neighboring the hilum and micropyle.

### 2.3. Osmopriming Caused Additional Void Spaces in Seeds

In a dry mature seed, the embryo is surrounded by the seed coat, leaving a few air-filled spaces between its outer surface and the testa. Removing the testa revealed more air-filled spaces between the cotyledons and embryonic axis in the primed dried seeds in comparison to the dry unprimed seeds (Figure 2C). Moreover, dry, unprimed seeds were significantly smaller in diameter than both the primed non-dried and dried seeds by 0.39 mm (16.8%) and 0.33 mm (14.5%), respectively (Figure 2B). The primed and unprimed seeds differed in size also during germination. The diameter of the primed seeds after 7 h of germination was significantly higher (11%) than the diameter of the unprimed ones.

### 2.4. Osmopriming Increases the Vacuolization Level of Cotyledon Cells

**Figure 6:**
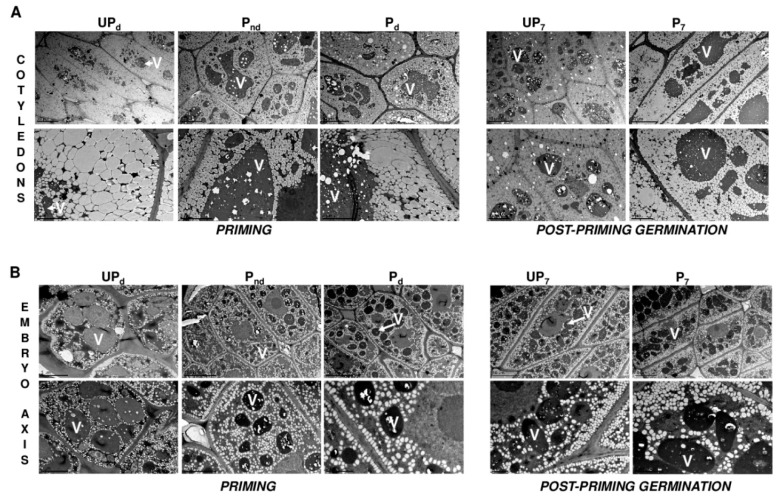
TEM micrographs showing the vacuolization level of the (**A**) cotyledons and (**B**) axis cells in unprimed and primed *B. napus* seeds before the start of germination (dry unprimed seeds, UP_d_; non-dried primed seeds, P_nd_; and primed dried seeds, P_d_) and during germination (unprimed seeds germinating 7 h, UP_7_; and primed seeds germinating 7 h, P_7_). The arrows indicate the vacuoles (V), and the bars represent 10 µm.

Figure 6A illustarates the ultrastructure of the cotyledon cells of the unprimed dry seeds, of the seeds after different stages of the priming treatment, as well as of the primed and unprimed seeds germinating for 7 h in water. In general, the main components of the cotyledon cells were oil bodies and protein storage vacuoles (PSV). The TEM views in Figure 6 should be regarded as a visual demonstration of the changes occurring in the protein and lipid reserve organizations during either priming or germination since no precise quantification of the protein storage vacuole and oil body sizes were performed. TEM analyses revealed that in the cells of each seed variant, the shape of the oil bodies varied, ranging from rounded or oval through elongated to angular. Also, the sizes of these organelles were diverse, and the most uniformly sized oil bodies were present in the non-dried primed seeds. The protein storage vacuoles were spherical or irregular in shape with a higher electron density than the oil bodies. The matrix of PSVs contained small intra-organellar inclusions similar to globoids.

Since almost the entire internal spaces of the cells were filled with storage materials, no other organelles were observed in the cotyledon cells of all the seeds apart from the nucleus with the electron-dense nucleolus. The most prominent differences between the primed and unprimed seeds were the size and number of PSVs in the cotyledon cells. The osmopriming treatment was associated with a reduction in the number of vacuoles and the increase in their volume. The greater degree of vacuolization was observed in the cotyledon cells of primed seeds germinating for 7 h in comparison to the unprimed seeds. In contrast to the unprimed seeds, few large vacuoles with minor deposits dominated in the primed seeds during germination.

No clear ultrastructural differences were evident between the axis cells of primed and untreated seeds, unlike that observed in the cotyledons (Figure 6B). The embryo axis cells of all the seed-derived samples were lower in lipids than the cells from cotyledons. The oil bodies of the embryo axis cells were more loosely scattered throughout the cytoplasm, with a tendency to accumulate along the cell membrane and to surround the other organelles. This pattern of oil bodies distribution in the cells was the most pronounced during germination of both the primed and unprimed seeds. The cells contained numerous small PSVs, spherical in shape and filled with globoids. The level of vacuolization of the axis cells was not noticeably different between the primed and unprimed seeds in either the dry state or during germination.

### 2.5. Osmopriming Alters the Expression Pattern of Aquaporin Genes

**Figure 7:**
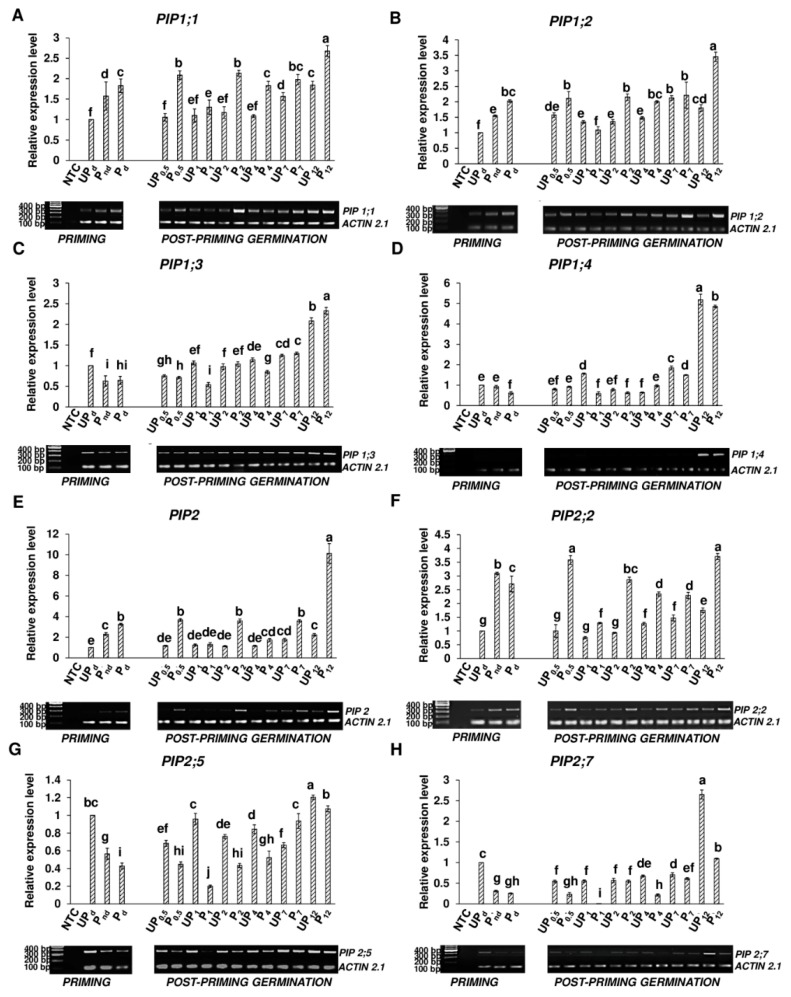
The semi-quantitative PCR analysis of the (**A**) *PIP1.1*, (**B**) *PIP1.2*, (**C**) PIP1.3, (**D**) *PIP 1.4*, (**E**) *PIP2*, (**F**) *PIP2.2*, (**G**) *PIP2.5*, and (**H**) *PIP2.7* gene expression levels in the seeds of *B. napus* before the start of germination (dry unprimed seeds, UP_d_; primed non-dried seeds, P_nd_; and primed dried seeds, P_d_) as well as during germination (unprimed seeds germinating at 0.5 h, UP_0.5_; 1 h, UP_1_; 2 h, UP_2_; 4 h, UP_4_; 7 h, UP_7_; and 12 h, UP_12_ and primed seeds germinating at 0.5 h, P_0.5_; 1 h, P_1_; 2 h, P_2_; 4 h, P_4_; 7 h, P_7_; and 12 h, P_12_). NTC, no template control. The differences were statistically significant as determined by the one-way analysis of variance (ANOVA) and the Tukey–Kramer Multiple Comparison Test (*n* = 6, *p* < 0.01). The same letters on the bars indicate insignificant differences between the means. The vertical bars denote SD.

Using the semi-quantitative PCR technique, the osmopriming-caused modulation of the expression pattern of genes encoding plasma membrane AQPs belonging to the *PIP1* and *PIP2* subgroups was investigated (Figure 7). To ensure the whole picture of the changes taking place during germination, transcript levels were examined at short intervals up to 7 h, i.e., after 0.5 h, 1 h, 2 h, 4 h, and 7 h and additionally after 12 h.

The *PIP1* subgroup (Figure 7A–D): Our results showed the accumulation of the *PIP1;1* and *PIP1;2* transcripts during the osmopriming treatment while the expression level of the *PIP1;3* and *PIP1;4* genes decreased during this treatment. The expression of both the *PIP1;1* and *PIP1;2* genes increased by 57% following the soaking phase of osmopriming and reached a higher level roughly by two times (by 83% and 102% respectively) after the redrying step as compared with untreated dry seeds. In the case of the *PIP1;3* gene, the transcript accumulation in the primed non-dried seeds was lower by 37% than in the unprimed ones and was maintained at this level in the primed dried seeds. The expression of the *PIP1;4* gene was not affected by PEG soaking but decreased significantly during the drying procedure. Considering the germination process of the primed seeds, the *PIP1;1* gene was significantly upregulated after the first 30 min of imbibition as compared to the primed dried seeds. Following a transitional reduction after 1 h and 4 h of germination, the expression level sustained until 7 h and maximized after 12 h of germination. For the unprimed seeds, the *PIP1;1* transcript accumulation did not change within the first 4 h of germination as compared to the dry seeds. The induction of the *PIP1;1* expression was observed after 7 h and 12 h of hydration, although the number of transcripts was significantly lower as compared with the primed seeds germinating for both 7 h and 12 h. The upregulation of the *PIP1;2* gene in the primed seeds was observed after 12 h of hydration. Prior to 12 h after hydration, the *PIP1;2* expression level was the same (at 0.5 h, 2 h, 4 h, and 7 h of germination) or lower (at 1 h of germination) as compared to the primed seeds in the dry state. Conversely, in the unprimed seeds, the *PIP1;2* gene activation was observed just after 30 min from the start of imbibition. Despite this, the expression level was generally lower than in germinating primed seeds (with exception to 1 h of germination when it was higher and 7 h when it was the same as in germinating primed seeds). The most prominent difference was noted after 12 h of germination when the accumulation of *PIP1;2* in the primed seeds was twice as high as in the untreated seeds. In the primed seeds, the expression of the *PIP1;3* gene declined after 1 h of water inflow, then started to gradually rise, and achieved maximum level after 12 h of germination when it was nearly thrice as high as in the dried seeds. For the unprimed seeds, the expression level initially decreased below the level in the dry seeds and then grew slowly, reaching the same level as in the dry seeds after 2 h. The expression of *PIP1;3* continued to increase and after 12 h of germination, the level was two times higher than in the dry seeds. During the germination of unprimed seeds, the expression of *PIP1;3* was the same or higher as compared with germinating primed seeds. However, it changed after 12 h of imbibition when the accumulation of the *PIP1;3* transcript in the primed seeds was statistically significantly higher by 12%. The expression of the *PIP1;4* gene in the primed seeds oscillated for the first 4 h of germination around the level in non-dried or dried primed seeds, then it strongly increased about 2.5 and 8 times after 7 h and 12 h, respectively, of germination. Similar trends were also observed in the unprimed seeds with a higher level of transcript accumulation by 12% and 15% after 7 h and 12 h of water uptake respectively as compared with germinating primed seeds.

The *PIP2* subgroup (Figure 7E–H): The expression of *PIP2* and *PIP2;2* were activated following osmopriming, in contrast to the *PIP2;5* and *PIP2;7* genes wherein expression declined during this process. The upregulation of the *PIP2* transcript started during the PEG soaking and continued during the drying phase. After the drying process, the *PIP2* transcript level increased up to a 3.25 times higher value as compared to the unprimed seeds in the dry state. The expression level of *PIP2;5* gradually decreased during the PEG soaking phase and drying process to reach a 2.3 times lower value in the primed dried seeds than in the unprimed dry seeds. Osmopriming was strongly associated with a reduced expression level of *PIP2;7* by roughly 3.2 times as compared with the unprimed seeds, particularly during the PEG soaking, since the drying process did not affect the expression of *PIP2;7.* For primed seeds, the accumulation of the *PIP2* transcript was preserved at the level of the primed dried seeds up to 7 h of germination with a transient drop after 1 h and 4 h. A high upregulation was observed after 12 h when the expression was elevated three times above the level in the primed dried seeds. For unprimed seeds, the accumulation of *PIP2* was maintained as in the dry unprimed seed until 4 h of germination and then slightly increased; however, it was 2 and 4.5 times lower after 7 h and 12 h, respectively, as compared to germinating primed seeds. In germinating primed seeds, accumulation of the *PIP2;2* transcript was higher than in the primed dried seeds after 30 min from the start of water uptake as well as after 12 h by approximately 35%. Between 20 min and 12 h following imbibition, the expression level of this gene was lower than in the primed dried seeds. For unprimed seeds, the number of transcripts was maintained as in the dry seeds until 2 h of germination and then began to increase, achieving a maximum value after 12 h of germination. Untreated seeds had a significantly lower accumulation level of *PIP2;2* for the whole time of germination compared with germinating primed seeds. The expression of *PIP2;5* in the primed seeds decreased two times after 1 h of germination as compared with primed seeds in the dry state and then gradually grew to reach a 2.5 times higher level after 12 h of germination than before this process. For unprimed seeds, the accumulation of the *PIP2;5* transcript during the first 7 h was lower or unchanged as compared with the dry unprimed seeds. After 12 h of germination, the expression of *PIP2;5* exceeded the level in the dry seeds by 20%. Generally, in the unprimed seeds, the level of the accumulated *PIP2;5* transcript during germination was notably higher than in the primed seeds. The highest *PIP2;7* expression level was observed after 12 h of imbibition for both germinating unprimed and primed seeds. The highest accumulation occurred in unprimed seeds; the expression level of *PIP2;7* after 12 h increased in the unprimed seeds roughly 2.5 times as compared with the unprimed dry seeds and was two times higher than in the primed seeds germinating for 12 h.

## 3. Discussion

The time course of water uptake by angiosperm seeds during germination includes three phases. Rapid initial water influx related to the passive imbibition of dry tissues associated with water movement first occurring in the apoplastic spaces represents phase I, which is followed by a period of reduction or even lack of water absorption called the lag phase (II), terminated by radicle protrusion. The last phase (III) incorporates a further sharp increase in hydration associated with embryonic axis elongation and seedling establishment. The results of this study showed that osmopriming with PEG greatly changes the kinetics of water uptake by *B. napus* seeds during germination. Osmopriming increased not only the rate of water uptake but also the amount of absorbed water (Figure 2A). Differences between the primed and unprimed seeds were visible after the first 15 min of imbibition. The successive increase in water content to certain thresholds during imbibition progressively recruits different types of biochemical and cellular events in germinating seeds. The first physiological activity restarted due to water absorption is respiration and amino-acid metabolism [22,43]. In *B. napus*, respiratory resumption begins in the residual endosperm and propagates in accordance with the route of water distribution inside the germinating seed [18]. Typically, phase I involves the subsequent initiation of other processes such as the translation of stored mRNA, de novo mRNA synthesis, and DNA repair, while upon phase II, regarded as a metabolism active phase, the start of vacuole enlargement, the mobilization of reserves, and embryo growth are observed [22,44]. Since the temporal pattern of events engaged in the preparation of the embryo to emerge is a function of the water content, faster and more effective hydration of the embryonic tissues caused by osmopriming may lead to a reduction of the germination time. In this paper, we demonstrated that the primed seeds of *B. napus* germinated nearly two times faster and more uniformly than the unprimed seeds (Figure 1). These results are consistent with our previous works, which showed that osmopriming promotes the germination of *B. napus* seeds both in optimal conditions and under salt stress [35,36]. Better germination parameters concomitant with the acceleration of seed imbibition were also recorded in germinating seeds of clovers primed with PEG and GA_3_ [38].

It should be noted that improved water uptake during post-priming germination cannot be considered as the only factor responsible for the enhanced germination of osmoprimed seeds. It has been found that one of the reasons responsible for the rapid germination of primed seeds is the initiation of germination-related processes during prehydration [45], which allows the primed seed to achieve a more advanced metabolic state before germination starts. Kubala et al. [35] established that during the soaking phase of osmopriming, *B. napus* seeds imbibed water up to 50%, which should be enough to reinitiate metabolism. Moreover, a comparative proteomic analysis of osmoprimed seeds and unprimed ones during germination showed changes in the abundance of different functional types of proteins [46], supporting the general view that the increased germination rate and efficiency of primed seeds results from the acceleration of early germination-related events as well as from switching on a set of mechanisms that alter the physiological state of the seed in a priming-specific manner. Kubala et al. [35] suggested that during post-priming germination, the production of proteins related with a faster germination was a direct consequence of preliminary priming. Nevertheless, the authors did not rule out that proteins accumulated during certain germination times of the primed seed may appear later in the unprimed seeds when they will reach a similar water content. Taken together, superior germination of primed seeds is an outcome of many overlapping mechanisms induced by priming, including efficient water absorption which certainly has a significant contribution to this effect.

On the other hand, results showing that osmoprimed seeds of *Solanum lycopersicum* imbibed less water during phase I of germination than control seeds were obtained by Nagarajan et al. [47]. Similarly, a slower rate of imbibition together with the reduction of leakage conductivity were detected in sweet corn seeds invigorated via solid matrix priming [48]. The authors identified these changes as potential causes for the observed improvement in the germination of the primed seeds, allowing for the reduction of early imbibitional damage and improved reorganization of the membranes. Indeed, excessively rapid initial water uptake can lead to a so-called imbibitional injury of the seed, primarily manifesting as a disturbance to the structural integrity of the membranes and resulting in the leakage of the soluble cellular contents [49]. The relationship between the rate of hydration and occurrence of imbibitional damage has been confirmed in seeds of different species [50,51,52]. The results from this study suggests, however, that the increase in water uptake by *B. napus* osmoprimed seeds does not entail excessive imbibitional damage as the germination potential was improved. 

In this study, a detailed water status characterization of germinating *B. napus* seeds was performed through NMR spectroscopy and spin–spin relaxation time (*T*_2_) measurement. Several previous NMR experiments demonstrated that *T*_2_ in hydrated seeds is characterized by 3 components corresponding to different water proton systems with varying mobility [16,47,53,54]. Based on the relationship that the shorter the relaxation times of the protons, the lower degree of molecule mobility, the results of these studies suggest that the population of short relaxation time protons corresponds to the bound water, a thin layer surrounding and hydrating macromolecules; protons with average relaxation times are associated with less mobile water while protons with long relaxation *T*_2_ times are associated with mobile (free) water. The study by Garnczarska et al. [16] indicated that the reorganization of water inside the swelling lupine seed during the early stages of imbibition occurred by a marked increase in structural/bound water and decrease in the other components. In this study, the presence of four clearly separate proton pools with different mobilities were observed (Figure 3). In addition to three water fractions identified as mobile, less mobile, and bound water, the fourth pool of protons was recognized as an oil fraction. While the differences between primed and unprimed seeds in the course of curves representing individual water fractions during swelling were negligible, prominent changes were visible in the case of proton systems representing lipids reserves. During early imbibition (the first 2 h from the start of hydration), a decline of *T*_2_ values over time associated with oil was more rapid in the primed seeds, indicating faster water penetration into the seed and more efficient tissue hydration in comparison with the unprimed seeds. Moreover, the abundance or pool of the water for each of the three components was also determined through the fit. Data that concern the contribution of particular fractions in the total pool of water pointed out that the dominant component during the imbibition of *B. napus* is the population of bound water (Figure 4). A strong increase for bound water (until about 1 hour after the start of hydration) was observed both in the primed and unprimed seeds. The value of contribution increased faster in the primed seeds, suggesting that during the initial period of imbibition, hydration of the biomolecules was more efficient in the seeds subjected to the osmopriming treatment than in the control. The changes in the *T*_2_ components during imbibition were investigated also in the primed and unprimed seeds of tomato [47]. The authors pointed out that, in contrast to our findings, hydration water was not detected in the dry state of primed and control seeds. However, hydration water appeared during imbibition faster in primed seeds than in unprimed seeds. This behavior allowed the authors to suggest that the better performance of the primed seeds may be related with an increased pool of hydration water, which seems to be confirmed in our results. 

In order to explore the priming-induced mechanisms leading to a more favorable water status of germinating seeds, the comparison of the seed coat anatomical traits of primed dried and unprimed dry seeds was conducted. SEM analysis revealed physical differences between pretreated and control seeds in the structural features of the seed coat. The coats of the primed were less undulate then those of the unprimed seeds, which may be interpreted as a seed coat expansion process caused by hydration and swelling of the seed during the soaking phase of the priming treatment (Figure 5). Secondly, seed coats of osmoprimed seeds were marked by multiple microcracks located mainly inside the pits that were only slightly visible in the case of the seed coats of the unprimed seeds (Figure 5D). The seed coat acts as a physical barrier that envelopes the embryo and modulates the movement of water into the seed. Some published experimental data indicate that the occurrence of cracks provokes a change in the water-permeable properties of the seed coat that can affect the imbibition process considerably. In permeable cultivars of soybean (soft seeds), the cuticle covering the palisade layer of the seed coat possessed minute cracks, whereas in impermeable cultivars (hard seeds), the cuticle was intact, demonstrating that the cracks are the routes of water entry into the seed [8]. The presence of small cracks on the seed coat surface as one of the major factors related to seed coat permeability was proposed also by Vu et al. [55]. Furthermore, Ma et al. [8] revealed that the distribution of cracks coincides with the sites of initial water uptake. A similar relationship may apply in the case of osmoprimed *B. napus* seeds. In the present study, we documented that the microcracks were formed only in the seed coat area adjacent to the hilum and micropyle regions (Figure 5), whereas it has recently been proved that the entry point of water in *B. napus* seeds is a seed coat section neighboring the hilum [18]. In this regard, the development of microcracks and their specific emplacement on the seed coat of primed *B. napus* seeds may considerably change the permeability of the seed coat and, therefore, be a crucial feature of the osmopriming treatment that triggers the enhancement of seed hydration during post-priming imbibition. Modifications of the seed coat structure and not in the form of cracks, however, but as circular depressions and seed coat tears were detected in the seeds of *Trifolium repens* subjected to the priming pretreatment, potentially favoring seed hydration [38]. In this study, the observed microcracks likely originated on account of the hydration–dehydration process during osmopriming, which generates tension of the seed coat and can lead to the disruption of its certain layers. The validity of this assumption is supported by an experiment in which the soaking of intact soybean seeds for an hour in water and then redrying resulted in the development of cracks [56]. Nevertheless, the question of why microcracks appear during osmopriming only in a specific seed coat area of *B. napus* seeds remains open. It can be presumed that it is an effect of the higher susceptibility of this region to stress and cracks formation due to its distinct local properties such as thickness or strength.

Osmopriming, specifically seed soaking, caused swelling of the seeds and increased the seed size that was maintained even following seed drying (Figure 2B). Upon removing the seed coat in the dried seeds, additional void spaces were observed inside the primed seeds (Figure 2C), probably as a result of the preliminary hydration followed by a loss of moisture and embryonic tissue shrinkage occurring during seed redrying. The development of internal free spaces via osmopriming increased the contact area between the water entering the inside of the seed during imbibition and the individual embryonic organs, thus facilitating cellular water flow and accelerating tissue hydration and expansion. X-ray photographs showed the presence of free space between the cotyledons and radicle of clover seeds invigorated by osmo- and hormopriming that was not observed in the seeds of the control [38]. The void space detaching the embryo and endosperm induced by osmo- and hydropriming was demonstrated in tomato seeds, and the link between this structural change and superior germination was also suggested [57,58,59].

In this study, we also investigated whether seed priming induces any ultrastructural changes in the axis and cotyledons of *B. napus* seeds (Figure 6). Radicle protrusion results from the cell elongation process. The initiation of this process requires additional water infiltration and is directly preceded by the mobilization of reserve proteins as well as the transformation of the protein storage vacuoles (PSVs) into lytic vacuoles (LVs) [60]. Vacuoles, as a specialized cell compartment accumulating osmotically active solutes, are involved with water influx to the cells from the walls leading to hydrostatic pressure development and cell swelling and are one of the elements regulating the hydration status in germinating seeds. It should be emphasized that activities associated with germination such as hydration, breakdown of the storage proteins inside PSVs, and cell vacuolization do not appear in all embryonic tissues in parallel, but they follow tissue-specific patterns [16,19,61]. Kuraś [62] indicated that in *B. napus* seeds, the first zones that are metabolically activated during germination are the root-cap columella cells and the basal part of hypocotyl, from which activation spreads in waves to the whole embryo. It was shown that in germinating *Arabidopsis thaliana* seeds, cell elongation starts in the lower hypocotyl and hypocotyl–radicle transition zone [21]. The developmental and biochemical processes leading to germination completion depend on water availability and begin earlier in the axis than in the cotyledons [29,63]. It would be expected that the osmopriming of *B. napus* seeds would be associated with a change in the axis ultrastructure since it is the first organ (particularly the radicle) to be hydrated [18]. Surprisingly, we have not observed any obvious ultrastructural differences between the axial cells of primed and untreated seeds, either in the dry seeds or during swelling (Figure 6B). However, osmopriming induced faster vacuolization in cotyledons by reducing the amount of PSVs with a simultaneous sharp increase in their volume, which was evident in both dry seeds as well as in the germinating ones (Figure 6A). Cotyledons constitute the biggest organ of a *B. napus* embryo, and they are the main recipients of water, hence the higher cells vacuolization of these organs can significantly contribute to the improved water status of the primed seeds. A possible explanation for earlier priming-induced vacuole enlargement in cotyledonary cells with no changes in the axis might be the altered architecture of the primed seed by the appearance of an additional free space in the central part of the seed that would potentially allow for easier and faster water inflow to the cotyledons. Munz et al. [18] showed that individual organs extend differentially during germination, and the highest increase in volume occurs in the case of inner cotyledons; therefore, the swelling of this embryo component provides a mechanical force supporting the seed coat rupture by the radicle. In the context of these findings, it is possible that osmopriming, despite the absence of a significant influence on the cellular elongation of the axis, facilitates the seed coat rupture and ultimately germination completion by the acceleration of cotyledons expansion. 

At the question of a higher water flow rate and hydration level during the germination of primed seeds as compared with the unprimed ones, it was interesting for us to investigate the expression level of the genes encoding PIP aquaporins—proteins that play a key role in the regulation of water transport across cell membranes. The upregulation of several *PIP* genes during germination was observed in *B. napus* seeds by Ge et al. [28]. In rice, the overexpression of the *OsPIP1;1* gene enhanced the germination rate in optimal conditions and the overexpression of the *OsPIP1;3* promoted germination under water stress [26,27]. Moreover, the distinct expression pattern of 11 *PIP* genes suggests that specific isoforms of PIPs could play different roles in seed germination [26]. The results of studies conducted on *Arabidopsis* indicated that AQPs (both PIP and TIP) are not involved in early seed imbibition but rather are associated with embryo growth and facilitate water supply to expanding tissues [25].

To this date, only a few attempts have been made to determine the potential role of water transport via AQPs in the enhanced germination rate of primed seeds. Gao et al. (1999) linked the superior germination of primed *B. napus* seeds with the induction of *BnPIP1* as a gene functionally related to the water transportation required for the enzymatic metabolism of storage nutrients at the early stages of seed germination. Chen et al. [40] indicated the *SoPIP1;1* and *SoδTIP* genes as the most related to the germination enhancement of primed spinach seeds. In our previous work, we detected an increase in the transcript abundance of *TIP4.1* and *TIP1.2* during the *B. napus* seed priming procedure as well as approximately a 74-fold higher expression of *TIP1.2* after 7 h of post-priming germination as compared to germinating unprimed seeds [34]. In this study, we examined the expression pattern of 8 *PIP* genes, and our results showed that the priming treatment modified the expression of all the tested genes (Figure 7). In both the primed and control seeds, the expression of all eight genes was detected in dry seeds; however, the priming treatment significantly altered their transcripts levels: in the cases of *PIP1;1, PIP1;2 PIP2,* and *PIP2;2*, there were priming-depended gene upregulation while others were downregulated. Besides *PIP1;3*, a similar tendency was observed during germination, i.e., the genes with expressions that increased in the dry seeds as a result of the priming treatment were also characterized by a higher level of expression in the primed seeds during germination as compared to germinating control seeds, and vice versa. Since priming leads to a more efficient water absorption and improved *B. napus* seed germination, we postulate that the priming-specific pattern of the *PIP* gene expression during germination documented in this study constitutes one of the reasons priming has a beneficial effect on water relations. However, considering the changes in the PIP gene expression as a function of germination time, separately for the primed and control seeds, the level of transcript accumulation at the early stages of hydration were around the values determined in the dry seeds and the most significant increase usually appeared already after 12 h of germination. Since, for both the primed and unprimed seeds, 12 h of hydration is sufficient to exceed the T_1_ time (Figure 1B), the above described general trend suggests that the water transport through plasma membranes by PIP channels potentially plays a greater role in the final stage of *B. napus* germination as well as during very early seedling growth than at the beginning of seed hydration. The most evident changes in gene expression after 12 h of germination both in relation to the output level of the transcript in dry seeds and between germinating primed and unprimed seeds were observed in the case of *PIP1;2* (within the *PIP1* subgroup) and *PIP2* and *PIP2;7* (within the PIP2 subgroup). Therefore, the distinct alternations induced by seed priming in the expression of these genes after 12 h of germination (i.e., strong upregulation of *PIP1;2* and *PIP2* while maintaining a relatively low level of the *PIP2;7* transcripts as compared to unprimed seeds) may have a crucial role in governing effective water transport from cell to cell in the expanding tissue during the late phase of germination of *B. napus* seeds and after radicle protrusion. Although a direct link cannot be made between the presence of AQPs transcripts and AQPs activity, it was suggested that PIP1 and PIP2 proteins do not act redundantly but have distinct functions in changing the genetic programs and differ in water permeability [64]. Concomitantly, changes in the expression of *PIP1s* and *PIP2s* genes might lead to a change in particular AQP isoform proportions and thus modulate the AQP tetramers composition. It is also possible that during germination and the reactivation of metabolism, the turnover and reorganization of AQPs take place. Particular forms of PIP1s and PIP2s could be also relocated and specifically localized in different tissue and/or organs within the germinating seed. It is premature to conclude the exact function of a particular form of PIP1 and PIP2; however, the present results provide a basis to a better understanding of the integrated functions of AQPs in germination and their role in enhancing the response to seed priming.

## 4. Materials and Methods 

### 4.1. Seed Osmopriming Treatment, Germination Conditions, and Experimental Arrangement

The seeds of *Brassica napus* L. cv Libomir were obtained from the OBROL company. Before the priming treatment, the seeds were surface sterilized in 70% ethanol solution for 5 min followed by incubation in a 1% sodium hypochlorite solution for 15 min. After sterilization, the seeds were rinsed with sterilized deionized water and air-dried for 15 min. Seed priming was conducted with polyethylene glycol 6000 solution with an osmotic potential of −1.2 MPa in darkness for 7 days at 25 °C as described previously by Kubala et al. [35,36]. After the priming treatment, the seeds were carefully rinsed with sterilized deionized water in order to remove the osmotic agent and then redried to the original water content (5%) for 48 h at room temperature. The primed dried (P_d_) and dry unprimed (UP_d_) seeds were stored at 4 °C until use.

The germination of P_d_ and UP_d_ seeds was carried out in darkness at 25 °C. The seeds were placed in plastic Petri dishes lined with three layers of filter paper moistened with 10 mL of deionized water. A seed was considered germinated when the radicle protruded the seed coat. To investigate the performance of unprimed and primed seed germination, the following parameters were measured: mean germination time (MGT), uniformity of germination (U9010: time interval of between 10% and 90% of viable seeds to germinate), and time to reach 1% of germination (T_1_) using “Germinator curve-fitting1.27.xls” Microsoft Excel script [65] and the mathematical approach described by El-Kassaby et al. [66]. Germination tests were carried out on ten replicates of 100 seeds.

In all experiments, UP_d_ seeds (control) and P_d_ seeds were used. Additional seed-samples were used in the analysis of the ultrastructure and measurement of the seed diameter, i.e., primed seeds collected before redrying to the original water content (P_nd_, primed non-dried) as a first crucial point of osmopriming, primed seeds germinated for 7 h (P_7_), and unprimed seeds germinated for 7 h (UP_7_). The imbibition time of 7 h corresponds to the achievement of a 1% germinated primed seed. The experimental layout of an AQP gene expression assay has been further expanded by unprimed and primed seeds germinating at 0.5 h (UP_0,5_, P_0,5_), 1 h (UP_1_, P_1_), 2 h (UP_2_, P_2_), 4 h (UP_4_, P_4_), and 12 h (UP_12_, P_12_).

### 4.2. The Kinetics of Water Uptake by Germinating Seeds

The kinetics of water uptake by unprimed and primed seeds during germination was determined by the gravimetric method. The seeds were allowed to imbibe in darkness at 25 °C in plastic Petri dishes containing three layers of filter paper wetted with 10 mL of deionized water. The seed samples were collected after selected intervals of hydration, blotted dry, weighed, and dried at 80 °C to constant weight. The water content after each specific time was calculated using the following equation: Three replicates of 0.3 g seeds for each experimental variant were used.

water content = W_2_ − W_1_/W_1_ (1)

where W_2_ is the weight of the seeds after hydration (g) and W_1_ is the dry mass of the seeds (g). The change in the moisture of the seeds was expressed as the grams of water absorbed per 1 gram of dry weight. 

### 4.3. Seed Diameter Measurement

The diameter of the unprimed seeds (UP_d_), seeds during the crucial time-points of the priming process (i.e., at the end of the soaking period before dehydration (P_nd_) and after the dehydration step (P_d_)), as well as germinating unprimed and primed seeds (UP_7_ and P_7_, respectively) was measured. The analysis was performed using the ImageJ 1.51 K software. The diameter was measured between the micropyle and the cotyledon region, located opposite to the micropyle. Ten replicates of 100 seeds each were used.

### 4.4. Nuclear Magnetic Resonance Analysis

Nuclear Magnetic Resonance (NMR) investigations were carried out at room temperature using a Bruker Avance DMX spectrometer operating at 400 MHz. The Carr–Purcel–Meiboom–Gill (CPMG) sequence was used to measure the transverse relaxation time *T*_2_ [67]. The time between echoes was 500 μs; however, only even echoes from the total amount of 4196 were taken into account for the data analysis. Before the experiment, the seeds were allowed to imbibe and germinate in darkness at 25 °C in plastic Petri dishes lined with three layers of filter paper moistened with 10 mL of deionized water. After selected hydration times, the seeds were collected, gently wiped with a dry filter paper, and then placed in a tube. In order to have more statistically reliable data, the experiment was repeated three times with 0.3 g seeds each for every stage of hydration. 

As the decay of the spin echo signal clearly showed a multi-exponential nature, a DISCRETE algorithm for a discrete sum of exponential decays [68,69] was used to calculate particular *T*_2_ relaxation times. Based on the obtained results, it was found that four *T*_2_ relaxation times can be distinguished which are attributed to various protons populations.

### 4.5. Scanning Electron Microscopy

Scanning electron microscopy (SEM) was used to examine the seed coat microstructure of the primed dried and dry unprimed seeds. The seeds were mounted directly on the SEM stubs using special SEM stage conductive carbon double-sided tape and sputter coated with approximately 30 nm thickness of gold. The specimens were examined and photographed using a Jeol 7001TTLS Scanning Electron Microscope at an accelerating voltage of 10.0 kV in high vacuum conditions and a working distance of 6.1 mm. The experiment was repeated three times.

### 4.6. Transmission Electron Microscope

The preparation of the embryo axes and cotyledons for transmission electron microscopy (TEM) was performed as described by Reference [70]. The isolated seed embryo axes and cotyledons were fixed in a half-strength Karnowsky’s fixative: a mixture of 2% paraformaldehyde and 2% glutaraldehyde [71]. Post-fixation was carried out in 1% OsO4. The samples were stained in a 2% aqueous solution of uranyl acetate. Dehydration was achieved using a graded series of acetone solutions. The objects were embedded in epoxy resin of low viscosity [72]. Ultrathin cross-sections were cut using Reichert Ultracut S () (Leica, Austria). The grids were post-stained in 5% uranyl acetate and 0.5% lead citrate and observed under the transmission electron microscope Jeol JEM 1400. The experiment was repeated tree times.

### 4.7. Expression Levels of Aquaporin’s mRNAs: RNA Isolation, cDNA Synthesis and Semi-q PCR Conditions

The total RNA was extracted according to the procedure described by Reference [73]. The removal of DNA contamination from the samples was performed using RQ1 (RNA Qualified) RNase-Free DNase (Promega, Leiden, The Netherlands), following the manufacturer’s instructions. The qualitative and quantitative assessment of RNA was carried out spectrophotometrically using NanoDrop ND-1000 (Isogen Life Science, DeMeern, The Netherlands) by verifying the absorbance ratios A260/280 and A260/230 (>1.8). RNA quality was further confirmed by agarose electrophoresis. Two µg of the total RNA of each sample were used for cDNA synthesis. Reverse transcription was performed using Revert Aid H minus first strand cDNA synthesis kit (Fermentas, St Leon-Rot, Germany), according to the manufacturer’s instructions. cDNA (200 ng) from each sample was used in the semi-qPCR experiments. The primers were designed using the NCBI/Primer-BLAST tool (www.ncbi.nlm.nih.gov/tools/primer-blast). Like previously [35,36], the *ACTIN2.1* gene (NCBI GenBank Accession: FJ529167.1; GI: 241740071) was selected as a reference gene for the purpose of normalization. Amplification was performed using DreamTaq Green DNA Polymerase (Fermentas, St Leon-Rot, Germany) under the following conditions: initial denaturation step at 95 °C, 4 min; thereafter 30 cycles consisting of denaturation for 45 s at 95 °C, annealing for 30 s at the preselected temperature depending on the primers used, and elongation for 25 s at 72 °C. The last step was the final extinction at 72 °C for 5 min. The preliminary experiments were conducted to identify a linear range of the initial amount of cDNA and the number of cycles ensuring that the performed PCR reactions were appropriate for quantitative analysis. The sequences of all the primers, the name of the target genes, the GenBank Accession Number, and the selected annealing temperatures are listed in Table 1. The PCR products were sequenced as described by Kubala et al. [36] to confirm that the designed primers flanked the target cDNA. The amplified products were then visualized on ethidium bromide-stained 1.8% agarose gels. The expression levels of AQP genes were determined by gel densitometry using GelixOne software and expressed as relative values compared to the actin expression (peak size of target gene/peak size of actin) and the addition ratio to the UP_d_ value. For each variant, the RNA was extracted from three biological replicates, and two PCR experiments were performed per replicate.

### 4.8. Statistical Analysis

Analyses were conducted at least in three independent experiments. Depending on the type of analysis, the statistical deviation of the mean values was calculated using one-way analysis of variance (ANOVA) and the Tukey–Kramer multiple comparison test or Student’s *t*-test. The means were considered as significantly different at *p* < 0.05, *p* < 0.01, or *p* < 0.001. 

## 5. Conclusions

**Figure 8:**
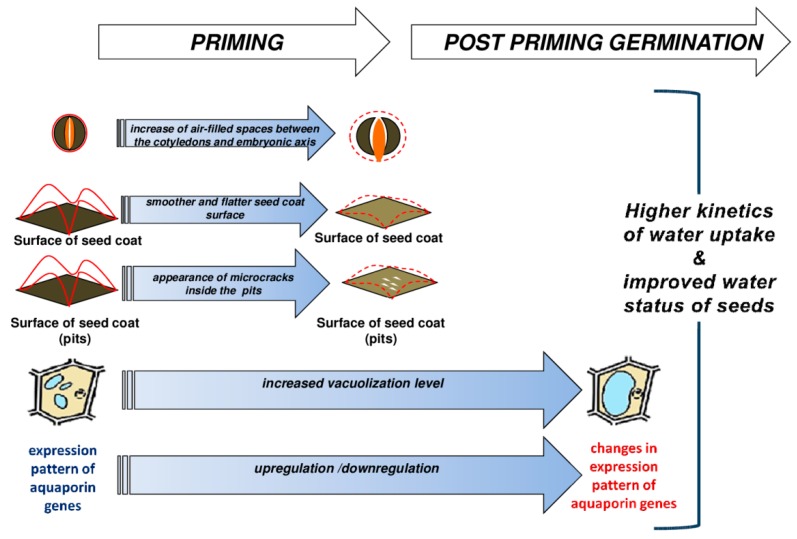
A schematic illustration of the osmopriming-induced factors affecting the kinetics of water uptake and, consequently, the water status of *B. napus* seeds during post-priming germination. The primed dried seeds are characterized by the presence of (i) more air-filled spaces between the embryo organs resulting in the bigger size of seeds and (ii) an altered seed coat structure, which facilitates faster and easier hydration of the seeds during post-priming germination. Moreover, during the priming procedure as well as during seed germination later, the enhanced vacuolization process of cotyledon cells and the modification of the gene expression pattern of the plasmalemma AQPs are observed.

In conclusion, the above findings are summarized in Figure 8 which represents a schematic illustration of the osmopriming-induced factors affecting the kinetics of water uptake, the amount of absorbed water, and consequently, the water status of *B. napus* seeds during post-priming germination. The primed dried seeds are characterized by the presence of more air-filled spaces between the embryo organs resulting in the bigger size of the seeds and an altered seed coat structure, which facilitates faster and easier hydration of the seeds during post-priming germination. Moreover, enhanced vacuolization of the cotyledon cells during the priming procedure as well as during seed germination later contributes to weaken the seed coat and thus facilitates subsequent radicle protrusion. NMR data suggest that the dominant component during the imbibition of *B. napus* is bound water. This bound water is mainly related to the cell walls. The involvement of apoplastic water movement and the diffusion of water across membranes seems interesting in regards to the stimulating effect of priming on the *PIP1;1, PIP1;2, PIP2*, and *PIP2;2* genes. It seems that water transport and sufficient water supply for embryo during germination may be one of the crucial components modulated by seed priming. Thus, it is important to continue research in the context of seed priming and aquaporin accumulation to identify the AQPs isoforms responsible for improved germination. 

## Figures and Tables

**Table 1:** A list of the plasma membrane intrinsic protein (PIPs) genes, the GenBank Accession Numbers, the primer sequences, and the annealing temperatures used in this study for the semi-qPCR purpose.

Gene	GenBank Accession Number	Forward Primer 5′–3′	Revers Primer 5′–3′	Annealing Temperature (°C)
*PIP1.1*	KF277205.1	CACTGTTTTGACCGTCATGG	TCCAAGACCACTTCCTTTGG	55
*PIP1.2*	KF277206.1	CTTGCTTCCTGGTCCTTCTG	GGCTCCACCTCCTAGAGCTT	55
*PIP1.3*	KF277207.1	CTTTCGGTGGCATGATCTTT	AGCGGAGAAGACGGTGTAGA	60
*PIP1.4*	KF277208.1	ACATCAGCTCAGTCCGACAA	CCTAGCCAAGAACAGACCGA	60
*PIP2*	AF118383.1	CGAGTTCGTAGCCACTCTCC	AACCGCTCTAACCAGCGATA	55
*PIP2.2*	KF277209.1	GTGACGTTCGGCTTGTTCTT	AGTGGCCAAGTGTACCATGA	55
*PIP2.5*	KF277210.1	CCCTTTACCCTGACCAGTGT	CGGAGAAGACGGTGTAGAC	60
*PIP2.7*	KF277211.1	CCCTTTACCCTGACCAGTGT	CGGAGAAGACGGTGTAGACT	55
